# Sleep, Affect, and Social Competence from Preschool to Preadolescence: Distinct Pathways to Emotional and Social Adjustment for Boys and for Girls

**DOI:** 10.3389/fpsyg.2017.00711

**Published:** 2017-05-23

**Authors:** Joan E. Foley, Marsha Weinraub

**Affiliations:** Family and Children's Research Collaborative, Department of Psychology, Temple UniversityPhiladelphia, PA, USA

**Keywords:** sleep problems, anxiety, depression, social competence, middle childhood, adjustment

## Abstract

Using a normative sample of 1,057 children studied across 4 waves over 6 years with multiple informants, we investigated transactional relations for sleep problems, anxious-depressed symptoms, and social functioning from preschool to preadolescence, assessing cumulative effects on children's emotional and social adjustment. To examine sex differences in the developmental processes, we conducted separate analyses for boys and girls. For both boys and girls, longitudinal cross-lagged panel analyses showed that preschool sleep problems directly predicted anxious-depressed symptoms 2 years later; indirect effects continued into preadolescence. For girls, early and later sleep problems directly or indirectly predicted a wide variety of preadolescent emotional and social adjustment domains (e.g., depressive symptoms, school competence, emotion regulation, risk-taking behaviors). For boys, social competence played a more important role than sleep problems in predicting preadolescent adjustment. Among the first set of findings that demonstrate longitudinal relations between sleep problems and social functioning in middle childhood and preadolescence, these results support Dahl's and Walker's neurological models of sleep and emotional functioning. We discuss these findings in light of relations between sleep and affect during pre-pubertal development and discuss differential findings for boys and girls.

## Introduction

Emerging research supports a role for children's sleep in the optimal regulation of affective functioning, yet this relationship is likely complex, bidirectional, and influenced by developmental change (Dahl, 1996; Walker, 2009; Leahy and Gradisar, 2012; Kahn et al., 2013; Kelly and El-Sheikh, 2013; Wang et al., 2016; Williams et al., 2016). For example, insufficient sleep appears to interfere with the capacity to regulate behavior and emotion, with increased risk for heightened anxiety, negative mood, impulsivity, and a compromised ability to respond appropriately to social stresses (Sadeh et al., 2002; Gregory et al., 2005, 2009; Dahl and Harvey, 2007; Alfano et al., 2009; Soffer-Dudek et al., 2011). In turn, emotional distress may heighten anxiety and increase bedtime ruminations with risk for delayed or disrupted sleep (Dahl, 1996; Bos et al., 2009; Kahn et al., 2013).

Relations between sleep and affect overlap due to the neurobiology central to sleep and the neurobehavioral systems underlying arousal and emotions (Dahl, 1996; Walker, 2009). In support of Dahl's (1996) model in which sleep is thought to optimize functioning of the prefrontal cortex (PFC), research shows that sleep deprivation interferes with the cognitive modulation of impulses, drives, and emotions governed by PFC (Horne, 1993; Giedd et al., 1999). Dahl suggests that the waking synchronization of multiple PFC-governed behaviors is particularly sensitive to sleep loss, and sleep loss may interfere with the complex processing required for effective social interaction. Walker's (2009) sleep and emotional information processing model holds that REM sleep, in conjunction with amygdala-hippocampal networks that encode salient events, promotes affective demodulation of previous affective experiences, and decreases their emotion intensity. Co-occurrence of sleep problems and anxiety and depression stems from the failure of demodulation and the negative ensuing consequences to emotional regulation (Gujar et al., 2011; Saletin et al., 2011). In support, research has demonstrated relations between children's sleep problems and depression during middle childhood (Costello et al., 2006). At least two-thirds of depressed children have considerable sleep onset and maintenance problems (e.g., Johnson et al., 2000; Bertocci et al., 2005). Taken together, difficulties with sleep and affect could have negative and spiraling effects over time on children's emotional and social adjustment. Such effects may lead to decrements in academic performance, feelings of isolation, clinical depression, substance abuse, and maladaptive risk-taking (Broidy et al., 2003; Shaw et al., 2003).

Less is known about how sleep, affect, and social competence influence one another during middle childhood. Middle childhood—ages 5–12 years (Broderick and Blewitt, 2003)—encompasses three facets of constitutional or contextual change: (1) the transition into formal school, (2) the transition to middle school, and (3) the period of pubescence when children begin to experience diverse and gradual biological and psychosocial changes associated with puberty. As children transition into formal schooling, expectations rise for self-control (McClelland et al., 2007). Dynamic and robust brain development during the preschool years results in considerable growth in higher order cortical regions like the prefrontal cortex that govern cognitive and behavioral control (Brown and Jernigan, 2012). Especially during critical periods of brain development, sleep disruptions may interfere with neuronal growth that aids in affect regulation (Dahl, 1996; Davidson, 2002; Ringli and Huber, 2011). Thus, disrupted sleep may compromise children's ability to demonstrate appropriate behaviors and emotions necessary for academic and social learning.

The end of middle childhood not only coincides with entry to more independent school settings (e.g., middle school), but it also heralds mounting biological, psychosocial, and environmental changes (Carskadon et al., 1980; Czeisler et al., 2005; Jenni and Dahl, 2008; Carskadon, 2011). The term “preadolescence” describes this period of rapid and extensive change when children typically transition from elementary to middle school. Because puberty appears to be trending earlier, by 9–12 years of age, and especially in girls (Herman-Giddens, 2006), pubertal changes to sleep and affect typically ascribed to adolescence (Carskadon et al., 2004; Carskadon, 2011) may also occur during this period. Recent evidence by Sadeh et al. (2009) appears to support this proposition. In support of this claim, Sadeh et al. (2009) demonstrated that pubertal changes to sleep (delayed sleep phase and disrupted sleep patterns) in children between 10 and 11 years *preceded* physical changes associated with puberty.

In preadolescence, changes in affect regulation coincide with risk for heightened emotional reactivity, depression, risky behaviors, and a greater need to align with peer group norms (Brown, 2004; Steinberg, 2008). Correlational studies have demonstrated decrements in mood, increased irritability, heightened sensitivity to negative stimuli, and ineffective coping strategies after experimental sleep restriction. Insufficient sleep and more sleep problems have been linked to increased risk-taking behavior (O'Brien and Mindell, 2005) with evidence for mediation through working memory processes (Thomas et al., 2015).

The current study builds on previous research examining the interplay between sleep problems and affective functioning (e.g., anxiety, depressive symptoms, and social competence) across middle childhood and the effects on preadolescent adjustment. Developmental models of self-regulation within a neurobehavioral framework (Dahl and Conway, 2009) guide our approach to uncovering the transactions that take place across a specific developmental period. Self-regulation refers to a set of processes and transactions that aid in regulating cognitive, affective, motor, and attentional control (Baumeister and Vohs, 2004). A neurobehavioral framework demonstrates the unique and complex neurological and behavioral insights into the development of interacting regulatory systems that underpin relations between sleep and affective functioning. Although Dahl and Conway focus on the effects of sleep loss on regulatory processes in adolescence, it is also meaningful to examine the effect of sleep difficulties and their interactions with self-regulatory processes during the period that precedes adolescence in light of findings by Herman-Giddens (2006) and Sadeh et al. (2009). By taking a longitudinal approach to the entire middle childhood period, we can examine the origins of these interactive influences and determine whether the interactions might in fact begin before adolescence. In the next section, we review the literature concerning the developmental course of regulation or risk for dysregulation in each of our domains of interest and the interactions that may exist between them in order to elucidate how biological, behavioral, and social ecological regulatory processes influence one another across middle childhood.

### Sleep problems and affective functioning across middle childhood

#### Sleep problems and self-regulation

After regulation of sleep-wake consolidation in infancy, development of the sleep-wake system reflects a slow and monotonic process evidenced by the disappearance of daytime naps (Donaldson and Owens, 2006), delays in bedtimes, and reduced overall sleep time (Wolfson, 1996). Sleep duration declines gradually with age during middle childhood although morning wake times remain relatively stable due to fixed school schedules (Biggs et al., 2013). At the same time, daytime sleepiness tends to increase and is more apparent in older children. Sleep quality appears to remain stable across the period even though overall sleep length declines. Developmentally, the continual and gradual increase in delayed sleep onset together with increasing daytime sleepiness has been associated with changes to the circadian system that occur during pubertal maturation in the preadolescent period (Sadeh et al., 2009). Findings by Sadeh et al. (2000) describe this ongoing and gradual change as “sleep-phase drift” in the period leading up to sixth grade.

Girls likely experience these circadian changes sooner than boys because pubertal onset for girls precedes that for boys. For example, Calhoun et al. (2014) concluded that increasing levels of insomnia symptoms in girls, but not boys, 5–12 years-of-age were likely related to hormonal changes associated with the peripubertal stage of development in girls. Prevalence of insomnia symptoms was highest and also peaked in girls ages 11–12 years. They found no differences between boys and girls in anxiety or depressive symptoms thus ruling out potential differences between boys' and girls' internalizing symptoms as a basis for differences in insomnia frequency. These findings demonstrate the importance of not only examining predictive relations between sleep and affective functioning during this period of transition, but also examining sex differences in their temporal relations.

Common sleep problems during middle childhood include night wakings, sleep onset delay from either constitutional or contextual factors, or insufficient sleep (Owens et al., 2000). Findings are mixed for the stability or change to night wakings during this period. Developmentally, night wakings are expected to decline with age; however, methodological differences may play a role in lack of consensus (Sadeh et al., 2000). Lack of agreement may stem from research suggesting less parental awareness of night time arousal in older children (Blader et al., 1997).

Conclusions regarding gender differences in sleep, beyond those associated with pubertal maturation, are limited and at times contradictory. Some studies suggest that girls sleep more and spend more time in motionless sleep compared to boys (Epstein et al., 1998) yet other studies have reported contradictory results (e.g., Gau and Soong, 1995). Enuresis is more common in boys than girls, declines over middle childhood, but is still present in a small percentage of boys at age 12 (Chandra et al., 2004).

### Sleep problems, anxiety, and depressive symptoms: regulating emotions

Anxious and depressive symptoms are subsumed under the overarching category of internalizing problems (Zahn-Waxler et al., 2000). They affect ~20% of youth at some point from childhood to adolescence, and have been associated with a series of negative outcomes such as academic failure, substance abuse, psychopathology, difficulties with social relationships, and suicide (e.g., Lewinsohn et al., 1993; Fergusson and Woodward, 2002). A review by Kovacs and Devlin (1998) suggested that anxiety disorders typically surface sometime during early to middle childhood (e.g., 6–11 years) and show moderate stability in childhood and adolescence (Gullone et al., 2001). In contrast, depressive disorders typically appear during early to middle adolescence (e.g., 11–15 years). Cole et al. (1997) further differentiated this progression with findings that anxiety symptoms and depressive symptoms around third grade were virtually indistinguishable in a normative sample. By sixth grade, each construct was distinct but highly related. Clark and Watson's tripartite model (1991) posits that the high co-occurrence for anxiety and depression emanates from a shared underlying component—negative affective temperament. Research has substantiated this claim in that difficult temperament and behavioral inhibition have been associated with risk for internalizing disorders (e.g., Biederman et al., 1990).

Recent evidence supports a competency-based model in the development of psychopathology asserting that a number of stage-salient skills or self-regulatory processes must be negotiated for competent development—for example, affect regulation, impulse control, social competence, and managing school transitions (Bosquet and Egeland, 2006). Failure to achieve competence in regulating one or more developmental processes may lead to internalizing disorders. At the same time, the development of internalizing disorders may interfere with attainment of competency. The authors examined this model by studying the etiology and course of anxiety symptoms from infancy through adolescence. They found that anxiety symptoms reflected moderate stability over the period. Heightened negative reactivity in infancy predicted emotion dysregulation in preschool which predicted anxiety symptoms in childhood. In turn, failure to regulate behaviors and emotion in childhood social interactions predicted anxiety symptoms in preadolescence, and anxiety symptoms in preadolescence predicted social incompetence in adolescence.

Buck and Dix's (2012) examination between development of depressive symptoms and peer relationships in early adolescence offers insight into why depressive symptoms may increase as children approach adolescence. Their findings suggested that youth who were less capable at regulating responses to physical and social stresses later in childhood were more likely to develop more depressive symptoms in adolescence.

In middle childhood, common sleep problems across various sub-categories of anxiety disorders include sleep onset latency, insomnia, nightmares, and nocturnal panic attacks (Gregory and O'Connor, 2002; Alfano et al., 2006; Kushnir and Sadeh, 2011; Kushnir et al., 2014) Overlap exists between the neurological processes required to self-regulate emotions and sleep. An increasing body of literature has concluded that the neurological circuitry for regulating emotions lies within the prefrontal cortex and the amygdala (e.g., Bechara et al., 1999). Yoo et al. (2007) found that sleep deprived individuals exhibited over 60% greater magnitude of amygdala reactivity in response to aversive stimuli compared to controls. Functional connectivity between the amygdala and the medial prefrontal cortex (mPFC) also showed significant reduction. Similar patterns of neuronal activity have been associated with anxiety disorders and depressive symptomatology (Davidson, 2002; Kent and Rauch, 2003).

Research has demonstrated that children who experience difficulty regulating their anxiety and fear at bedtime may also experience problems with falling asleep, potentially leading to bedtime resistance. Excessive parental involvement in managing bedtime resistance may adversely affect children's capacity to self-regulate both their sleep and emotion with risk for later developmental problems in both areas (Tikotzky and Sadeh, 2009). Mindell and Barrett (2002) reported increased levels of trait anxiety in 5–11 year old children with three or more nightmares per week. The ability to return to sleep without parental intervention after a nightmare requires the capacity to self-sooth and regulate. These findings demonstrate the importance of self-regulation in children's ability to manage the overlap between sleep and emotions. Evidence exists for developmental differences in relations between sleep and anxiety and sleep and depressive symptoms (Alfano et al., 2009). In prepubertal children, sleep and anxiety appear to be more closely related whereas sleep and depressive symptoms are more closely related in adolescents.

### Sleep problems and social competence: regulating behaviors and emotions

Social competence requires regulation of behaviors and emotions to forge friendships, build cooperation, and willingly participate in group activities (Bodrova and Leong, 2006). The developing ability to regulate attention control and inhibit responses, along with growth in cognitive processes such as working memory and planning functions, increase the child's ability to not only manage response strategies but to attend more constructively to social cues in engagement with family, teachers, and peers (Bronson, 2000; Blair, 2003). Achieving milestones on the path to social competence necessitates a series of transactions and dependencies involving motor, representational, and linguistic systems. Development of sensorimotor skills and growth in psychological processes such as attention, memory, language, and executive function alter arousal and response tendencies from infancy through middle childhood—all of which underpin and promote regulation of behavior and emotion in support of successful social interaction.

Children who demonstrate poor self-regulation are more likely to be rejected and ostracized by peers (Bierman et al., 2015). Consequently, the interplay of poor behavioral control and peer rejection creates a dynamic process highlighting the effects of individual differences in social-emotional functioning as early as elementary school (Miller-Johnson et al., 2002). Evidence has shown that social incompetence early in middle childhood predicted increased internalizing symptoms at the end of middle childhood (Gazelle and Ladd, 2003). Burt et al. (2008) reported similar associations during this period using a design capable of establishing directionality. Social competence in middle childhood showed negative longitudinal links to internalizing symptoms in adolescence in a normative sample; reverse associations and gender differences were not found. The authors concluded that findings were broadly consistent with developmental competency-based models of internalizing symptoms with important implications for positive adjustment in the future.

Developing social competence involves the interaction and integration of multiple regulatory systems that govern brain/behavior relations. Maturational processes within the brain and nervous system continue to develop into adolescence (Gunnar and Vazquez, 2006), and regions of the prefrontal cortex, associated with behavior and emotion regulation, are among the slowest maturing brain systems. Numerous parallels and overlap exist with sleep-related neurobiologic systems and the neurobehavioral systems that regulate affect, attention, learning and memory, and language (Dahl, 1996). Sleep is thought to optimize functioning of the prefrontal cortex (PFC), and research shows that sleep deprivation interferes with the cognitive modulation of impulses, drives, and emotions governed by PFC (Horne, 1993). Dahl suggests that waking synchronization of multiple PFC-governed behaviors is particularly sensitive to sleep loss, and sleep loss may interfere with the complex processing required for effective social interaction.

During the preschool years and beyond, girls demonstrate better social competence due to lower prevalence of externalizing behaviors, better effortful control and language development, and less physical aggression (Zahn-Waxler et al., 1995; Kochanska et al., 2000; Broidy et al., 2003). Evidence suggests such behaviors are relatively stable across development and thus contribute to stability in levels of social competency over time (Masten, 2005; Burt et al., 2008).

Two studies of children's sleep problems examined relations between sleep and social competence. Vaughn et al. (2015) found that sleep duration was positively correlated with social competence and receptive vocabulary. Lemola et al. (2011) reported that sleep quantity and quality were related to optimism and self-esteem, but no significant associations surfaced between sleep and social competence.

### Sleep problems, affect, and preadolescent adjustment

Pubertal onset has been closely linked to changes in affective domains such as increased emotional intensity, sensation seeking, risk-taking, sleep/arousal regulation, and affective disorders (Herman-Giddens, 2006). It appears likely that affective changes, typically ascribed to adolescence and the teenage years, may occur somewhat earlier as puberty may occur somewhat earlier (Carskadon, 2011). Thus, we use the term “preadolescence” to isolate this period of rapid and extensive change when children typically transition from elementary to middle school. In light of these emerging trends, it is important to consider relations between sleep, affective functioning, and children's adjustment immediately prior to the teen years.

Biological and psychosocial changes that take place during this time manifest themselves through increased stress responsiveness and emotional reactivity (Dahl and Lewin, 2002; Nelson et al., 2005; Steinberg, 2008). Although this period may represent unique opportunities for learning and developing social and emotional skill, it also presents a time of considerable risk. In accordance with previous sections of this paper that reviewed the literature on children's psychological functioning and sleep problems across middle childhood, children with feelings of maladjustment in preadolescence may be more likely to report lower levels of self-esteem, feeling less connected and competent in school, and more depressive symptoms. A dramatic increase in the extent of time spent with peers coincides with heightened emotional reactivity, depression, sensation-seeking, risky behaviors, and a greater need to align with peer group norms (Gardner and Steinberg, 2005; Steinberg, 2008). Evidence has shown considerable overlap among these constructs such that biological and environmental risk factors may explain why some children develop maladaptive behaviors leading to poor emotional and social adjustment. Insufficient sleep, more anxious and depressive symptoms, and poor social competence have shown unique associations with poor social and emotional adjustment during the transition to adolescence. Less clear, however, are the interactions among these domains across middle childhood and their cumulative effects on preadolescent functioning.

Children's feelings of competence and well-being are the product of the dynamic interplay of academic and social experiences that have taken place over a lengthy period of childhood (Parker et al., 1995; Ladd and Burgess, 2001). Developmental differences are evident in children's perception of academic competence (Stipek and Daniels, 1988; Wigfield et al., 1997). In early elementary school, young children typically overestimate their academic abilities as demonstrated by lack of convergence with teacher ratings of performance (Stipek and Iver, 1989). By second or third grade, however, closer alignment begins to exist between *children's perspectives* and *objective indices* of performance (Wigfield et al., 1997). By the time children reach the end of middle childhood, their feelings about themselves as students have had sufficient time to form. The adaptive or maladaptive feelings that children develop at the end of middle childhood begin to form the basis for coping with broader academic and psychological adjustments associated with impending school transitions.

By adolescence, coping styles and strategies to regulate emotions have likely influenced by both biological and environmental factors. Emotion regulation is based on the premise that “emotion is a multifaceted phenomenon involving physiological arousal, neurological activation, cognitive appraisal, attention processes, and response tendencies” (Thompson, 1994, p. 30). It reflects a developmentally changing phenomenon due to its reliance on biological and neurological processes. Similar to temperament models, individual differences illuminate why emotional arousal and selection of emotion response strategies lack uniformity and consistency across individuals (Thompson et al., 2011). Development of sensorimotor skills and growth in psychological processes such as attention, memory, language and executive function alter arousal, and response strategies from infancy through middle childhood (Harman et al., 1997; Zelazo and Cunningham, 2007).

Emotional dysregulation has been related to poor social functioning, internalizing and externalizing behavior problems, and maladaptive risk-taking (Aldao et al., 2016). Because sleep deprivation has been associated with emotional processing, it is not surprising that some correlational studies have demonstrated decrements in mood, increased irritability, heightened sensitivity to negative stimuli, and ineffective coping strategies after experimental sleep restriction. Less clear, though, is the cumulative effect of interactions between sleep and affect on emotion regulation across middle childhood.

As children approach adolescence, personal responsibility for judgment and decision-making increases while adult oversight of both academic and social behaviors gradually diminishes over time. Deciding to engage in maladaptive risk-taking behaviors stems from a breadth of factors including temperament, neurobehavioral disinhibition, social competencies, parenting, and peer behaviors (Feldstein and Miller, 2006). Risk-taking in the preadolescent period has been referred to as lack of attention to personal safety, stealing, cheating, property destruction, and substance abuse. A recent study by Thomas et al. (2015) has linked sleep disturbances (a factor in PFC performance) to maladaptive risk-taking, largely mediated by working memory processes. O'Brien and Mindell (2005) also found evidence for insufficient sleep and increased risk-taking behavior in adolescence. Tarter et al. (2004) have referred to neurobehavioral disinhibition as a composite of executive cognitive functioning, emotional regulation, and behavioral control with each function governed by the prefrontal cortex (PFC).

Longitudinal evidence across this period of middle childhood in normative samples (Fredriksen et al., 2004; Gregory et al., 2005; Alfano et al., 2009) suggests that sleep problems precede symptoms of anxiety and depression with relations beginning as early as school entry. These studies tested mostly unidirectional associations between domains and did not follow children across middle childhood. More recently, Kelly and El-Sheikh (2013) identified reciprocal relations between sleep and internalizing behaviors in children ages 8–13. Using a cross-lagged research design capable of identifying bidirectionality between domains, they found bidirectional effects with sleep having stronger effects on behavioral adjustment (e.g., internalizing and externalizing behaviors) than the reverse associations. In a prior study using a similar sample, El-Sheikh et al. (2012) reported similar associations with relations more pronounced for girls. Using a longitudinal cross-lagged model, Gregory et al. (2009) reported that sleep problems at 8 years predicted symptoms of depression 2 years later but reverse associations were not found. Wang et al. (2016) most recently found that emotional difficulties at age 5 differentiated normal sleepers from troubled sleepers over almost a decade, but the groups did not differ in emotional functioning in late adolescence. Even though gender was not significantly related to the initial level of sleep problems, girls with more early anxious and depressed symptoms were more likely over time than boys to be troubled sleepers. These studies lend insight into relations between sleep and affect during middle childhood and illustrate the importance of studying boys and girls separately. The limited concordance in findings, however, leaves open questions about directionality over extended periods of development, pathways of risk for emotional and social adjustment, and the potential for gender differences.

### Current study

The current study investigates the series of transactions that take place for sleep, anxiety and depressive symptoms, and social competence in a large community sample of children from preschool to fifth grade using a four-wave design capable of uncovering the direction of effects (Selig and Little, 2012) among these domains and their cumulative effects on emotional and social adjustment at the end of middle childhood (e.g., preadolescence). We chose a 6-year interval—between 4 ½ and 10 ½ years-of-age—because it covers all of middle childhood as well as two important transitionary periods—the start of formal schooling during which time children learn to adapt to new academic and social demands—and the transition to adolescence, a sensitive period of physical, cognitive, emotional, and social development. We defined sleep problems as night awakenings that disrupt sleep (including having nightmares, talks or walks in sleep, or wets the bed), sleeping less or more than typically developing children, difficulty initiating or maintaining sleep, and daytime overtiredness. We use the term *preadolescent adjustment* to encompass multiple aspects of emotional and social development that have been associated with sleep problems and affective functioning such as the ability to control emotional reactivity, levels of depressive symptoms, feelings about school, and engagement in maladaptive risk-taking behaviors. Our study used multiple informants, controlled for covariates associated with sleep and affect, and we examined effects separately by gender. Several studies have suggested that biological changes to sleep during pubertal development may result in different findings for boys and girls (Angold et al., 1998; Herman-Giddens, 2006; Sadeh et al., 2009; Carskadon, 2011).

**Figure 1:**
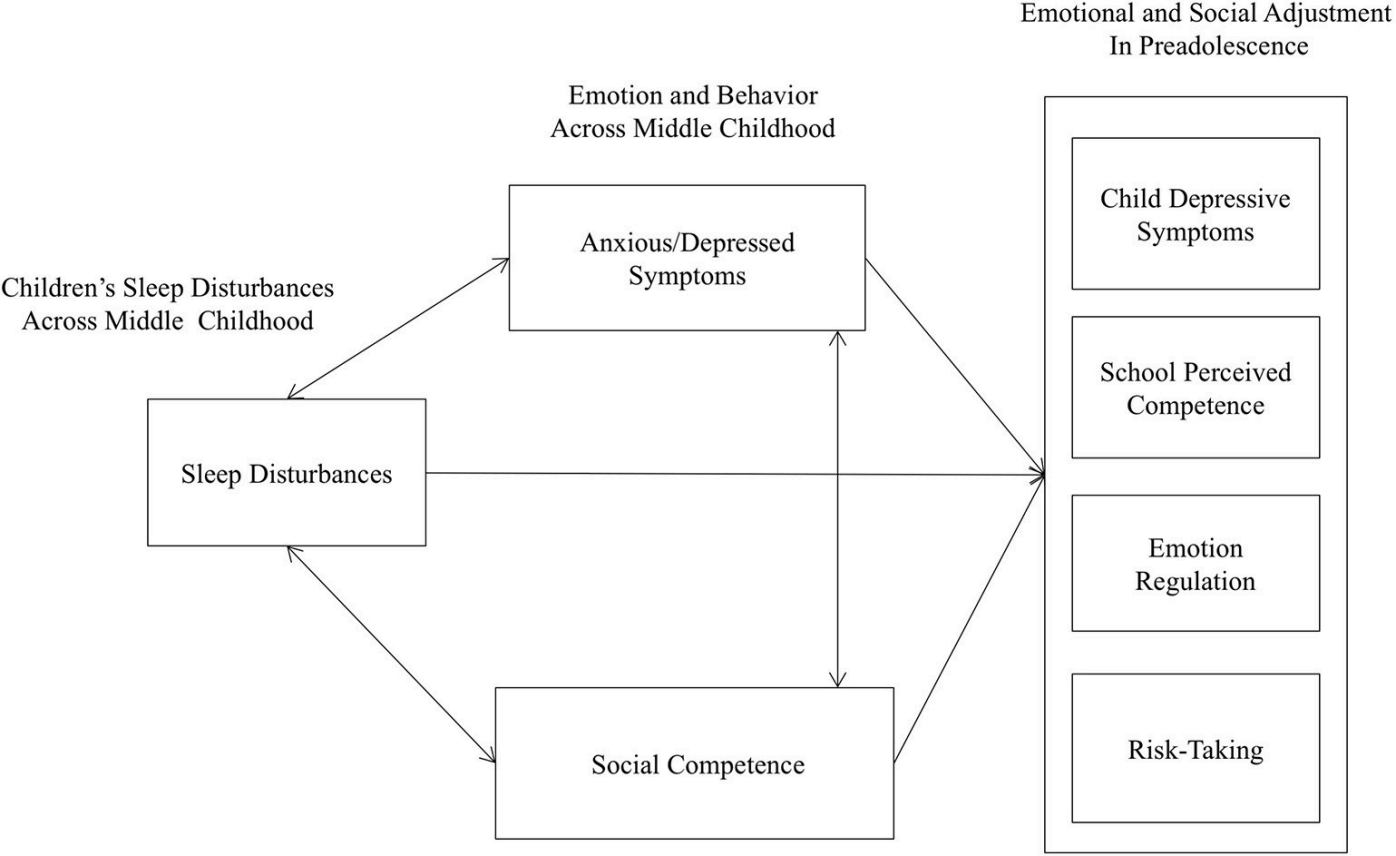
**Conceptual model of sleep, affect, and social competence across middle childhood and effects on preadolescent adjustment**.

Figure 1 reflects a conceptual model that hypothesizes bidirectional relations among domains across middle childhood to predict emotional and social adjustment during the transition to adolescence. This model depicts the interplay of multiple domains that may result in effects deemed as direct and unidirectional, direct and bidirectional, or indirect through various pathways, with each successive stage considered a partial product of previous stages and the current stage. Similar to developmental cascade models (Masten and Cicchetti, 2010), this model suggests that transactions that occur among domains over time may have adaptive or maladaptive cumulative effects on later functioning. Because cascade models typically focus on an entire developmental period, they have the potential to uncover periods of risk that may inform preventative intervention.

Finally, to rule out the possibility that any relations over time are due to shared risk factors (Coulombe et al., 2010) we included controls for maternal depressive symptoms (Warren et al., 2006; Seifer, 2011; El-Sheikh et al., 2012; Weinraub et al., 2012), difficult child temperament (Van Tassel, 1985; Sadeh et al., 1994; Atkinson et al., 1995; Owens-Stively et al., 1997; El-Sheikh and Buckhalt, 2005; Weinraub et al., 2012), marital instability (El-Sheikh and Kelly, 2011; Mannering et al., 2011), lax parental control (Owens-Stively et al., 1997; Noble et al., 2012), and lower socio-economic status (Grandner et al., 2013; Singh and Kenney, 2013).

We addressed five specific questions: (1) how are domains at adjacent time points from preschool to fifth grade related, (2) what is the magnitude of these relations and the pathways of influence to preadolescent functioning, (3) does early functioning *directly* predict later functioning over and above their indirect effects, (4) are there cross domain indirect effects from transactions across middle childhood, and (5) are there differences in these relations for boys and girls? Because research has focused limited attention on relations among all of the domains of interest and spanned the entire period of middle childhood, this study is exploratory.

## Materials and methods

### Sample

With a large and diverse sample of families from 10 different locations across the United States, the National Institute of Child Health and Development Study of Early Child Care and Youth Development (NICHD-SECCYD) publicly available longitudinal data set was ideal for testing our model. Details of study recruitment and protocols are described at http://www.icpsr.umich.edu. NICHD-SECCYD researchers recruited 1,364 families in 1991 through hospital visits with mothers giving birth (*n* = 8,986) during selected 24-h intervals in 10 locations in the United States. Written informed consent was obtained from all participants—parents and teachers. Proper assents were also obtained from the children at each age. The sample used in this report consisted of families in which mothers reported children's sleep problems, beginning at 54 months old, the initial time point for analysis (*n* = 1,057), and excluded those families where the informant for sleep was listed as “other” (*n* = 7). The sample was diverse, including 24% ethnic minority children, 11% mothers who had not completed high school, and 14% single mothers. At the start of the study, mothers had an average of 14.4 years of education (*SD* = 2.49 years); 51.7% of children were boys. The present report focused on children beginning at 54 months-of-age (*M* = 4.5, *SD* = 0.23) with assessments proceeding in 2-year, equal intervals (Grade 1, Grade 3, Grade 5) to provide a sufficient time lag for effects to take place. In Grade 1, children were 6.5 years of age; in Grade 3, children were 8.5 years of age, and in Grade 5 children were 10.5 years of age. There were no differences in ages between the boys and the girls.

### Procedure

Children and their mothers came to the university laboratories when children were 54 months old and when children were in Grades 1, 3, and 5. At these visits, standardized assessments were administered to the children and mothers. Teachers completed questionnaires regarding the study child's academic and social development from kindergarten through Grade 5. Children completed questionnaires about their social and emotional functioning in Grade 5.

### Measures

Table 1 depicts the timing of measurement for each predictor, outcome, and control variable used in the study.

**Table 1:** **Study variables and timing of assessment by grade**.

**Variable**	**Preschool (54 months)**	**Grade 1**	**Grade 3**	**Grade 5**
Sleep problems	√	√	√	√
Anxious/depressive symptoms	√	√	√	√
Social competence	√	√	√	√
Child depressive symptoms				√
School competence				√
Emotion regulation-teacher				√
Emotion regulation-mother				√
Risk-taking-mother				√
Risk-taking-child				√
**COVARIATES**				
Negative affective temperament	√			
Maternal depression	√^*^			
Socio-economic status	√^*^			
Marital quality	√			
Firm parental control	√			

* *Variable measured 6 times from 1 to 54 months*.

### Predictor measures—54 months, grade 1, grade 3, and grade 5

Predictor variables were measured repeatedly at the four time points listed above.

#### Sleep problems

Sleep problems that may reflect insufficient or inadequate sleep encompass a wide range of problems, from night awakenings that disrupt sleep (such as nightmares, talks or walks in sleeps, or wets the bed), sleeping less or more than typically developing children, difficulty initiating or maintaining sleep, and daytime overtiredness. Seven items on The Child Behavior Checklist (CBCL 4–18; Achenbach, 1991) specifically assess the incidence of these problems—nightmares, talks or walks in sleep, wets the bed, sleeps less than most kids, sleeps more than most kids, trouble sleeping, and overtired. The CBCL is a widely-used measure of children's behavior problems and has been used frequently in sleep studies where parental report served as the method to assess sleep problems (Gregory and O'Connor, 2002; Alfano et al., 2009; Friedman et al., 2009; Wang et al., 2016). Mothers rated each item as (2) very true or often true, (1) somewhat or sometimes true, or (0) not true as far as you know. For this study, we summed each of these items to identify children with more or fewer sleep problems. Higher scores reflected more sleep problems. Scores at each assessment could range from 0 to 14 though the observed range was from 0 to 9. The test-retest reliability of the CBCL measure was obtained by examining the correlation of maternal CBCL scores across assessments. They showed high reliability (0.49–0.54).

The CBCL Cronbach's alpha ranged from 0.50 to 0.56 over four assessments. However, for this measure, inter-item reliability was not relevant. It is only useful in measures for which the researchers are trying to tap an underlying construct. With the CBCL, we were not assuming an underlying sleep problem construct. Instead, we were trying to identify the different kinds of problems children might have with sleep and the cumulative frequency of problems. This CBCL measure simply indicates that children with higher scores have more sleep problems.

Becker et al. (2015) examined the convergent, discriminant, and external validity of the individual CBCL sleep items and a CBCL composite with validated measures of sleep functioning that included the Children's Sleep Habits Questionnaire (CSHQ), the Sleep Disorders Inventory for Students (SDIS), the Adolescent Sleep-Wake Scale (ASWS) as well as criteria used for diagnosing children's sleep disorders in 383 youth ages 6–18 years. As in this study, they summed the seven sleep-related items on the CBCL and found that the composite score exhibited very low internal consistency (Cronbach's alpha = 0.28). Because this score is consistent with prior research, Becker and colleagues concluded that a total score may still be “meaningfully related to other validated sleep measures that also tap multiple (and at times unrelated) domains of sleep functioning” (p. 81). They reported that the CBCL sleep composite was significantly and moderately-to-strongly correlated with the CSHQ total score, the ASWS total score, and several indices of the SDIS, and that the CBCL sleep composite may be optimal when examining overall sleep functioning and external correlates of sleep. In particular, Becker et al. proposed that the CBCL composite may be especially useful in large-scale studies and archival datasets where objective measures may be too intensive and costly for wide-scale use, such as this one.

#### Anxious and depressed symptoms

Mothers completed the CBCL Anxious-Depressed Symptoms Subscale at each assessment with responses of *not true, somewhat or sometimes true, or very true or often true* (Cronbach's alpha = 0.80–0.81). Examples of the 14 scale items included feeling sad, worries, feels worthless, nervous, fearful, guilty, and lonely. The test-retest reliability showed moderate reliability (0.45–0.62).

#### Social competence

At 54 months, teachers/caregivers completed the first 30 items from the California Preschool Social Competency Scale (Levine et al., 1969) and four additional items by Clarke-Stewart specifically for the NICHD-SECCYD (Cronbach's alpha = 0.88). Items focused on the child's cooperative play, rule following, empathy, personal safety, and aggression. Each item contained four descriptive statements (numbered 1–4) and ordered by increasing levels of competence relative to the behavior being measured. At Grades 1, 3, and 5, teachers completed a 10 item scale created for the NICHD-SECCYD study based on Gresham and Elliott's (1990) Social Skills Rating System (SSRS)-Peer Competence Scale (Cronbach's alpha = 0.87). For each item, teachers were asked “*How often*” using the following response levels: 0 = *Never*; 1 = *Sometimes; and* 2 = *Very Often*. Examples of the 10 items included controls temper in conflict situations, responds appropriately to peer pressure, and cooperates with peers without prompting. Higher scores indicated a higher positive peer response. The test-retest reliability showed moderate reliability (0.28–0.38).

### Outcome measures: preadolescent emotional and social adjustment—grade 5

#### Children's depressive symptoms

Children completed the short form of the Children's Depression Inventory (CDI, Short Form; Kovacs, 1992) designed to assess their level of depression (Cronbach's alpha = 0.74). Ten sets of three statements were presented and the child selected the one that best described the way she or he felt over the last 2 weeks. The items tapped dysphoric mood, lack of pleasure, and low self-esteem. Higher scores indicated more child depression.

#### Children's feelings about themselves in school

Children completed a 20-item questionnaire about school commitment which asked how they felt about school and themselves in school. The questionnaire incorporated 7 items directly from Eccles' Michigan Study of Adolescent & Adult Life Transition Questionnaire with other items from the Iowa Youth and Family Project conducted by the Institute for Social and Behavioral Research at Iowa State University. The School Total Score (Cronbach's alpha = 0.85) included questions related to the child's perceived competence in school, school motivation, and feelings about the social aspects of school. Children were asked how true each statement was for them using the following response levels: *1* = *Not true at all; 2* = *Not very true; 3* = *Sort of true; and 4* = *Very true*. Higher scores indicated more positive feelings about school.

#### Emotion regulation

Mothers and teachers responded to a 10-item questionnaire to measure perceptions of how children express emotions. Respondents rated their child's frequency of display of emotion on a 5 point scale: 1 = *Never*, 2 = *Occasionally*, 3 = *About Half the Time*, 4 = *Usually*, and 5 = *Always*. The questionnaires were identical for mothers (Cronbach's alpha = 0.77) and teachers (Cronbach's alpha = 0.83). Higher scores indicated more emotional reactivity. Teacher and mother scores were not combined to permit independent reporting.

#### Child risky behavior

Mothers and study children completed a questionnaire designed to assess the child's risky behaviors. The questionnaires differed in the number of items, item content, and response scales. Mothers completed a 30-item questionnaire that included items related to minor risk-taking (e.g., personal safety, cheating/lying/stealing by child, physical aggression, carrying weapons, and substance abuse). The response scale measured the frequency with which the child engaged in these behaviors: 1 = *Never*, 2 = Once or Twice, 3 = *More than two times*. A response of 0 = *Don't Know* was recoded to special missing values before creating any composite scores and all non-missing items were recoded to 0/1/2 to be comparable with the child-reported risky behaviors items. Higher scores indicated more risk taking behavior (Cronbach's alpha = 0.72). The child-reported 19-item questionnaire was adapted from the work of Conger and Elder (1994), the Fast Track Project, and the New Hope Project. The child was asked how often they engage in behaviors such as skipping school, fighting, smoking, and destroying property. Possible responses included 0 = *Never*, 1 = *Once or twice*, and 2 = *More than twice*. A response of 3 = *Refuse or Don't Know* were recoded to special missing values and all non-missing items were recoded to 0/1 (0 = *Never*, 1 = *Ever*). Higher values indicated more risk-taking (Cronbach's alpha = 0.65).

### Control variables

#### Child temperament-negative affectivity

Mothers completed the short form of the Children's Behavior Questionnaire (CBQ) (Rothbart et al., 1994) at 54 months-of-age containing 80 items and 8 scales. Three scales comprised the dimension of Negative Affectivity: fear, anger-frustration, and sadness (Cronbach's alpha = 0.64, 0.76, and 0.60, respectively). Each of these scales consisted of 10 items. We created a Negative Affectivity score for each participant by summing the results of the three scales (Cronbach's alpha = 0.60). Items were scored on a seven-point Likert scale that ranged from 1 = “*Extremely untrue” to* 7 = “*Extremely true*” and included a “*not applicable” response* category.

#### Maternal depressive symptoms

Mothers completed the Center for Epidemiological Studies (CES-D; Radloff, 1977) questionnaire when children were 1, 6, 15, 24, 36, and 54 months old. Because of the demonstrated enduring effects of early maternal depression (Goodman and Gotlib, 1999), we summed the maternal depression scores from six prior assessments of maternal depression from birth to the child's 54 month birthday. Cronbach's alphas for the CES-D across the six data collection points ranged from 0.85 to 0.91. The depression scores were moderately stable over time (*rs* = 0.46–0.58).

#### Marital quality

When children were 54 months-of-age, mothers who were living with a husband or partner or who reported having a long term relationship completed a questionnaire about marital quality. The Personal Assessment of Intimacy in Relationships (PAIR; Shaefer and Olson, 1981) reflected 36 items to assess the degree of intimacy an individual perceives with his or her partner. We used a 6-item subscale of the PAIR, Love, and Relationships, designed to measure the mother's intimacy with the spouse or partner. Mothers responded to a 5-point Likert scale with responses that ranged from *1* = “*Strongly disagree” to 5* = “*Strongly agree.*” Cronbach's alpha = 0.86.

#### Firm parental control

When children were 54 months-of-age, mothers completed a questionnaire designed to assess their discipline strategies with children. The questionnaire was an extensive revision of Greenberger's Raising Children Checklist (Shumow et al., 1998). The Firm Control subscale included 6 items related to praise and explanations for parental rules. Mothers responded *1* = “*Definitely No” to 4* = “*Definitely Yes.*” Higher scores denoted firmer, more responsive control (Cronbach's alpha = 0.60).

#### Socioeconomic status

We computed an income-to-needs ratio by using the parents' income, the number of individuals the mother reported in the household, and the U.S. census poverty levels for the time of measurement. A ratio <1 is considered “poor.” Because of enduring effects of low socioeconomic status on children's health and behavior (Bradley and Corwyn, 2002), we summed the results of six prior assessments of families' income-to-needs ratio from birth to the child's 54-month birthday.

### Data analytic strategy

The primary analyses used structural equation modeling (SEM) in Amos™ 19.0 (Arbuckle, 2010) to establish the relations and pattern of effects among the variables in the conceptual model Bentler (1988). Given the longitudinal nature of the data, some participants had missing values. We used full information maximum likelihood estimation (FIML) to address missing data in order to avoid omitting participants. FIML produces less biased estimates than either mean substitution or list wise case deletion (Graham, 2009; Acock, 2012).

Data analyses proceeded in seven stages and followed procedures similar to Little (2013) and Masten et al. (1995) for testing longitudinal cross-lagged panel models. For the entire sample, first we tested a model that examined stability between repeated measures (autoregressive model) from one assessment period to the next to examine rank order among participants over time while controlling for across and within-time covariation (Model 1). Second, we added cross-lagged paths between adjacent time points to test for bidirectional relations and the magnitude of effects among the domains across four waves of data (Model 2). Third, to test whether *early effects* of sleep problems, anxious-depressed symptoms, and social competence in middle childhood would exert *direct* effects on these same domains later in middle childhood (above their indirect effects), we added direct paths to Model 2 from each 54 month and Grade 1 domain to each domain at Grade 5 (Model 3). We removed (trimmed) all non-significant paths to improve parsimony. Then, we added a set of variables associated with preadolescent emotional and social adjustment to Model 3 to uncover the direct and indirect effects across middle childhood for sleep problems, anxious-depressed symptoms, and social competence (Model 4). Next, we added covariates to Model 4 using the semipartial control method (Little, 2013) to examine their predictive influence on the domains and the potential for moderating effects, and to determine whether relations among domains changed after inclusion of covariates (Little et al., 2007; Model 5). Then, we conducted a multiple group analysis based on Model 4 to examine whether relations differed for boys and girls before adding covariates (Model 6). Next, we added covariates to Model 6 (using the semipartial control method) to examine the influence of covariates in the gender model (Model 7). Last, we assessed the cross-domain overall indirect effects on outcomes across middle childhood and on preadolescent adjustment.

We used multiple criteria to examine how well the data fit the model. First, we assessed the chi-square index. A non-significant chi-square reflects a good fit between the observed covariances and the covariances implied by a fitted model. As Kline (2011) notes, chi-square is sensitive to sample size and may reflect statistical significance even though a model fits the data well when sample sizes are large, as in the present study. We also examined the root mean square of approximation (RMSEA; Steiger and Lind, 1980), the Tucker-Lewis index (TLI; Tucker and Lewis, 1973), and the comparative fit indices (CFI; Bentler, 1990). The RMSEA represents the magnitude of the discrepancies between the implied and actual covariances. A value of zero indicates a perfect fit and a value of 0.05 is an indication of a close fit (Browne and Cudeck, 1992; Hu and Bentler, 1999). The TLI and CFI represent comparisons of the current model to a null model, estimating the amount of total information explained by the current model. Values >0.90 and 0.95, respectively, indicate a good fit (Hu and Bentler, 1999).

## Results

### Descriptive statistics: changes over time

For the entire sample, average sleep problem scores gradually and significantly declined from 54 months-of-age to Grade 3 (*M* = 1.41, *SD* = 1.51; *M* = 1.00, *SD* = 1.27; *M* = 0.87, *SD* = 1.25, respectively; *p* < 0.001), but tended to remain relatively stable between Grades 3 and 5 (*M* = 0.81, *SD* = 1.24 for Grade 5; *p* < 0.10). Mothers rated nightmares as the most frequent sleep problem at each time point. At 54 months and Grade 1, mothers considered enuresis and sleeping less than other children as the next most prevalent sleep problems. At Grades 3 and 5, talking and walking in sleep, sleeping less than other children, and overtiredness ranked highest after nightmares. Mean scores for children's anxious-depressed symptoms increased from 54 months-of-age to Grade 1 (*M* = 52.08, *SD* = 4.26, *M* = 52.77, *SD* = 4.84, respectively, *p* < 0.001), and social competence declined from Grade 1 to Grade 3 (*M* = 15.34, *SD* = 0.3.62; *M* = 14.91, *SD* = 3.87, respectively; *p* < 0.01).

Table 2 presents a comparison of descriptive statistics for boys and for girls for each variable. With the exception of sleep problems at 54 months, there were no sex differences on sleep. As expected for a community sample, a considerable number of mothers reported no sleep problems for their child at each assessment. Thus, the mean values for sleep problems reflected a “floor effect” that might imply that sleep problems were not a problem for most children. To address this potential interpretation, we categorized sleep problems into two groups with “0” = mothers rated their child as having no sleep problems, or “1” = mothers rated their child as having one or more sleep problems “sometimes or often,” we found that at 54 months, mothers reported that 71% of boys and 61% of girls experienced one or more sleep problems sometimes or often times. In Grade 1, this percentage declined to 54% for boys and 51% for girls. By third grade, the percentage dropped to 49% for boys and 45% for girls. By fifth grade, 41% of boys and 43% of girls continued to experience one or more sleep problems sometimes or often. Thus, almost 50% of boys and girls experienced one or more sleep problems at some point across middle childhood.

**Table 2:** **Descriptive statistics for study variables and covariates for boys and girls**.

**Variable**	**Boys**			**Girls**			***p***
	***n***	**M**	***SD***	***n***	**M**	***SD***	
Sleep problems 54 m	525	1.54	1.55	521	1.28	1.46	^**^
Sleep problems G1	488	1.07	1.31	495	0.94	1.22	ns
Sleep problems G3	459	0.91	1.26	486	0.84	1.24	ns
Sleep problems G5	452	0.83	1.29	486	0.79	1.20	ns
Anxious/depressed 54 m	527	52.08	4.32	527	52.08	4.20	ns
Anxious/depressed G1	489	52.88	5.26	497	52.65	4.38	ns
Anxious/depressed G3	461	53.09	5.20	488	53.21	5.27	ns
Anxious/depressed G5	455	53.17	5.65	481	53.01	5.07	ns
Social competence 54 m	351	102.89	14.29	376	106.99	12.76	^***^
Social competence G1	471	14.81	3.62	484	15.86	3.55	^***^
Social competence G3	432	14.13	4.09	459	15.64	3.51	^***^
Social competence G5	410	14.41	4.01	435	15.58	3.63	^***^
**ADJUSTMENT**							
Child depressive symptoms G5	454	1.20	1.85	479	1.37	2.07	ns
School competence G5	457	3.40	0.38	479	3.57	0.31	^***^
Emotion regulation-teacher G5	418	28.38	7.00	437	28.16	6.59	ns
Emotion regulation-mother G5	456	33.08	5.76	482	33.94	5.58	^*^
Risk taking-mother G5	453	7.23	4.65	461	5.91	4.12	^***^
Risk taking-child G5	448	3.64	2.81	455	1.95	1.54	^***^
**COVARIATES**							
Negative affective temperament	380	12.86	1.70	378	12.85	1.81	ns
Maternal depression	466	46.50	32.93	473	47.90	32.67	ns
Socio-economic status	461	18.70	13.19	477	19.48	14.10	ns
Marital quality	439	3.87	0.87	442	3.74	0.90	^*^
Firm parental control	522	21.43	1.71	526	21.47	1.67	ns

*p-value represents the degree of significance between means for boys and for girls*.

* *p < 0.05*,

** *p < 0.01*,

*** *p < 0.001. ns, non-significant*.

There were no differences between boys' and girls' anxious-depressed symptoms at any assessment. Teachers consistently rated girls' as more socially competent compared to boys at all assessments. Boys' exhibited more risk-taking behaviors and felt less competent in school compared to girls. Mothers reported more emotional reactivity in girls compared to boys in fifth grade. No differences surfaced between boys' and girls' self-reported depressive symptoms, or emotional reactivity in the classroom in fifth grade. We found no significant differences for control variables between boys and girls with the exception of marital quality. Boys' mothers reported less satisfaction with the quality of their martial relationships compared to girls' mothers.

Tables 3, 4 present bivariate correlations for each measure for boys and girls, respectively. For both boys and girls, all repeated measures were significantly and moderately to highly correlated over age. Also, concurrent associations between model variables indicated that more sleep problems were associated with higher rates of anxious-depressed symptoms and less effective interactions with peers at all assessments. Girls' sleep problems and anxious-depressed symptoms were significantly related to preadolescent adjustment more often than boys. Boys' social competence was significantly related to preadolescent adjustment more often than girls.

**Table 3:** **Bivariate correlations between study variables for boys**.

		**1**	**2**	**3**	**4**	**5**	**6**	**7**	**8**	**9**	**10**	**11**	**12**	**13**	**14**	**15**	**16**	**17**	**18**	**19**	**20**	**21**	**22**	**23**
1.	SleepD54	−																						
2.	SleepDG1	0.50	−																					
3.	SleepDG3	0.36	0.54	−																				
4.	SleepDG5	0.32	0.41	0.51	−																			
5.	ANXD54	0.46	0.26	0.21	0.26	−																		
6.	ANXDG1	0.32	0.36	0.23	0.18	0.45	−																	
7.	ANXDG3	0.33	0.27	0.37	0.20	0.38	0.51	−																
8.	ANXDG5	0.31	0.28	0.35	0.42	0.34	0.47	0.61	−															
9.	SocialC54	−0.13	−0.10^a^	−0.04^a^	−0.07^a^	−0.15	−0.09^a^	−0.11	−0.22	−														
10.	SocialCG1	−0.13	−0.10	−0.04^a^	−0.02^a^	−0.08^a^	−0.07^a^	−0.09^a^	−0.15	0.38	−													
11.	SocialCG3	−0.07^a^	−0.05^a^	−0.07^a^	−0.09^a^	−0.01^a^	−0.03^a^	−0.16	−0.18	0.30	0.35	−												
12.	SocialCG5	−0.06^a^	−0.13	−0.06^a^	−0.06^a^	−0.01^a^	−0.12	−0.16	−0.21	0.27	0.27	0.37	−											
13.	CDepG5	0.04^a^	0.05^a^	0.12	0.06^a^	−0.03^a^	0.11	0.11	0.13	−0.16	−0.08^a^	−0.16	−0.20	−										
14.	SchoolG5	−0.03^a^	−0.11	−0.05^a^	−0.12	0.02^a^	−0.06^a^	−0.12	−0.16	0.24	0.17	0.20	0.28	−0.44	−									
15.	ER-TG5	0.04^a^	0.06^a^	0.07^a^	0.11	−0.09^a^	0.03^a^	0.06^a^	0.09^a^	−0.07^a^	−0.14	−0.18	−0.35	0.10^a^	−0.08^a^	−								
16.	ER-MG5	0.14	0.17	0.15	0.12	0.07^a^	0.18	0.22	0.28	−0.10^a^	−0.09^a^	−0.13	−0.15	0.03^a^	−0.03^a^	0.20	−							
17.	Risk-MG5	0.18	0.17	0.07^a^	0.15	0.05^a^	0.11	0.13	0.21	−0.20	−0.11	−0.16	−0.25	0.02^a^	−0.25	0.17	0.22	−						
18.	Risk-CG5	0.02^a^	0.03^a^	−0.08^a^	0.01^a^	0.01^a^	0.07^a^	0.01^a^	0.00^a^	−0.02^a^	−0.22	−0.17	−0.22	0.13	−0.24	0.18	0.01^a^	0.21	−					
19.	NegAffTemp	0.23	0.22	0.17	0.11	0.27	0.26	0.28	0.15	−0.14	−0.03^a^	−0.01^a^	−0.10	0.21	−0.17	0.12	0.19	0.13	0.08^a^	−				
20.	MDep	0.23	0.21	0.17	0.10	0.23	0.25	0.25	0.20	−0.09^a^	−0.15	−0.24	−0.24	0.20	−0.15	0.11	0.18	0.24	0.12	0.20	−			
21.	SES	0.05^a^	−0.08^a^	−0.01^a^	−0.04^a^	0.02^a^	0.00^a^	0.02^a^	−0.02^a^	0.15	0.07^a^	0.13	0.23	−0.08^a^	0.13	−0.11	−0.04^a^	−0.19	−0.15	−0.01^a^	−0.30	−		
22.	MaritalQ	−0.19	−0.12	−0.10^a^	−0.00^a^	−0.20	−0.20	−0.25	−0.15	0.14	0.11	0.09^a^	0.03^a^	−0.05^a^	0.09^a^	0.01^a^	−0.02^a^	−0.13	−0.01^a^	−0.13	−0.46	0.10	−	
23.	FirmPCntrl	−0.08^a^	−0.02^a^	0.02^a^	−0.05^a^	−0.06^a^	−0.05^a^	0.00^a^	−0.01^a^	0.13	0.04^a^	0.03^a^	0.01^a^	0.04^a^	0.06^a^	0.02^a^	0.04^a^	−0.14	0.01^a^	−0.02^a^	−0.17	0.12	0.14	−

*SleepD, Sleep disturbances; AnxD, Anxious-depressed symptoms; SocialC, Social competence; CDep, Child Depressive Symptoms; School, School competence; ER-T, Emotion regulation-teacher reported; ER-M, Emotion regulation-mother reported; RiskM, Risk-taking mother reported; RiskC, Risk-taking child reported; NegAffTemp, Negative affective temperament; Slp&Temp, Moderation-Sleep and Temperament; SES, Socioeconomic status; MaritalQ, Marital quality; FirmPCntrl, Firm parental control. All correlation coefficients significant at p < 0.05 unless otherwise noted*.

a *Not significant*.

**Table 4:** **Bivariate correlations between study variables for girls**.

		**1**	**2**	**3**	**4**	**5**	**6**	**7**	**8**	**9**	**10**	**11**	**12**	**13**	**14**	**15**	**16**	**17**	**18**	**19**	**20**	**21**	**22**	**23**
1.	SleepD54	−																						
2.	SleepDG1	0.49	−																					
3.	SleepDG3	0.41	0.54	−																				
4.	SleepDG5	0.33	0.45	0.52	−																			
5.	ANXD54	0.38	0.22	0.19	0.14	−																		
6.	ANXDG1	0.33	0.33	0.21	0.17	0.51	−																	
7.	ANXDG3	0.29	0.32	0.44	0.30	0.43	0.58	−																
8.	ANXDG5	0.26	0.26	0.31	0.40	0.40	0.53	0.62	−															
9.	SocialC54	−0.18	−0.06^a^	−0.11	−0.11	−0.02^a^	−0.05^a^	−0.11	−0.10^a^	−														
10.	SocialCG1	−0.17	−0.09^a^	−0.09	−0.08^a^	−0.04^a^	−0.10	−0.14	−0.04^a^	0.28	−													
11.	SocialCG3	−0.13	−0.05^a^	−0.08^a^	−0.07^a^	−0.06^a^	−0.07^a^	−0.19	−0.08^a^	0.22	0.38	−												
12.	SocialCG5	−0.13	−0.07^a^	−0.13	−0.16	0.03^a^	−0.02^a^	−0.11	−0.09^a^	0.26	0.33	0.35	−											
13.	CDepG5	0.07^a^	0.15	0.14	0.10	0.04^a^	0.11	0.19	0.21	−0.16	−0.05^a^	−0.11	−0.20	−										
14.	SchoolG5	−0.15	−0.16	−0.18	−0.14	−0.03^a^	−0.12	−0.15	−0.18	0.16	0.17	0.19	0.27	−0.50	−									
15.	ER-TG5	0.06^a^	0.07^a^	0.12	0.07^a^	0.01^a^	0.04^a^	0.09^a^	0.14	−0.13	−0.06^a^	−0.11	−0.21	0.03^a^	−0.08^a^	−								
16.	ER-MG5	0.08^a^	0.12	0.12	0.18	0.12	0.22	0.25	0.31	−0.02^a^	−0.05^a^	−0.06^a^	−0.10	0.10	−0.03^a^	0.18	−							
17.	Risk-MG5	0.23	0.22	0.20	0.22	0.16	0.21	0.28	0.31	−0.17	−0.13	−0.14	−0.14	0.18	−0.24	0.03^a^	0.12	−						
18.	Risk-CG5	0.09^a^	0.11	0.02^a^	0.03^a^	0.05^a^	0.11	0.07^a^	0.05^a^	−0.17	−0.15	−0.13	−0.20	0.22	−0.30	0.16	0.09^a^	0.30	−					
19.	NegAffTemp	0.29	0.20	0.12	0.18	0.28	0.34	0.25	0.20	−0.19	−0.12	−0.09^a^	−0.06	0.08	−0.08^a^	0.06^a^	0.29	0.10^a^	0.05^a^	−				
20.	MDep	0.17	0.14	0.09^a^	0.16	0.28	0.24	0.28	0.24	−0.08^a^	−0.14	−0.15	−0.16	0.15	−0.11	0.05^a^	0.16	0.20	0.15	0.29	−			
21.	SES	−0.04^a^	−0.04^a^	−0.02^a^	−0.05^a^	−0.04^a^	0.04^a^	−0.04^a^	−0.05^a^	0.14	0.07^a^	0.14	0.20	−0.11	0.18	−0.03^a^	−0.01^a^	−0.19	−0.22	−0.02^a^	−0.27	−		
22.	MaritalQ	−0.13	−0.09^a^	−0.02^a^	−0.06^a^	−0.19	−0.11	−0.12	−0.15	0.04^a^	0.04^a^	0.03^a^	0.03^a^	−0.08^a^	0.06^a^	−0.03^a^	−0.13	−0.16	−0.05^a^	−0.11^a^	−0.37	0.10	−	
23.	FirmPCntrl	−0.03^a^	−0.07^a^	0.02^a^	−0.01^a^	−0.01^a^	−0.09^a^	0.08^a^	−0.05^a^	0.02^a^	0.04^a^	0.07^a^	0.02^a^	0.04^a^	0.08^a^	0.02^a^	0.07^a^	−0.10	−0.05^a^	−0.03^a^	−0.10	0.13	0.05^a^	−

*SleepD, Sleep disturbances; AnxD, Anxious-depressed symptoms; SocialC, Social competence; CDep, Child Depressive Symptoms; School, School competence; ER-T, Emotion regulation-teacher reported; ER-M, Emotion regulation-mother reported; RiskM, Risk-taking mother reported; RiskC, Risk-taking child reported; NegAffTemp, Negative affective temperament; Slp&Temp, Moderation-Sleep and Temperament; SES, Socioeconomic status; MaritalQ, Marital quality; FirmPCntrl, Firm parental control. All correlation coefficients significant at p < 0.05 unless otherwise noted*.

a *Not significant*.

### Relations for sleep and affect across middle childhood and effects on preadolescent adjustment for boys and girls

**Figure 2:**
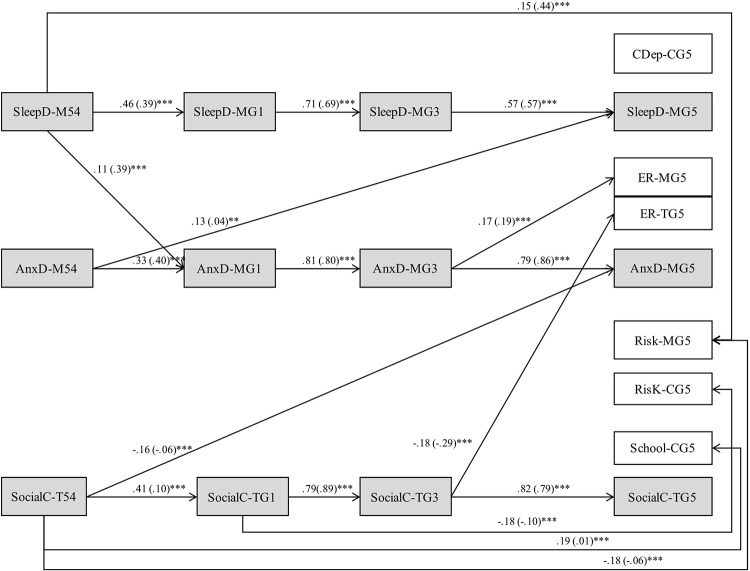
**Boys' final model depicting transactional relations across middle childhood and effects on emotional and social adjustment in preadolescence (***n*** = 528) after controlling for covariates and moderating effects**. Paths for covariance, residual covariance, and covariates, not shown for ease of presentation. Only significant pathways are displayed. Model fit: χ^2^ = 498.163; *df* = 324; *p* = < 0.001; CFI = 0.960; TLI = 0.930; RMSEA [90%] = 0.023 [0.019, 0.026]; Sleep D, sleep problems; AnxD, anxious-depressed symptoms; SocialC, social competence; CDep, child depressive symptoms; School, school competence; ER-M, emotion regulation maternal report; ER-T, emotion regulation teacher report; Risk M, child's risk-taking-maternal report; Risk C, child's risk taking-child report. ^*^p < 0.05, ^**^p < 0.01, ^***^p < 0.001.

**Figure 3:**
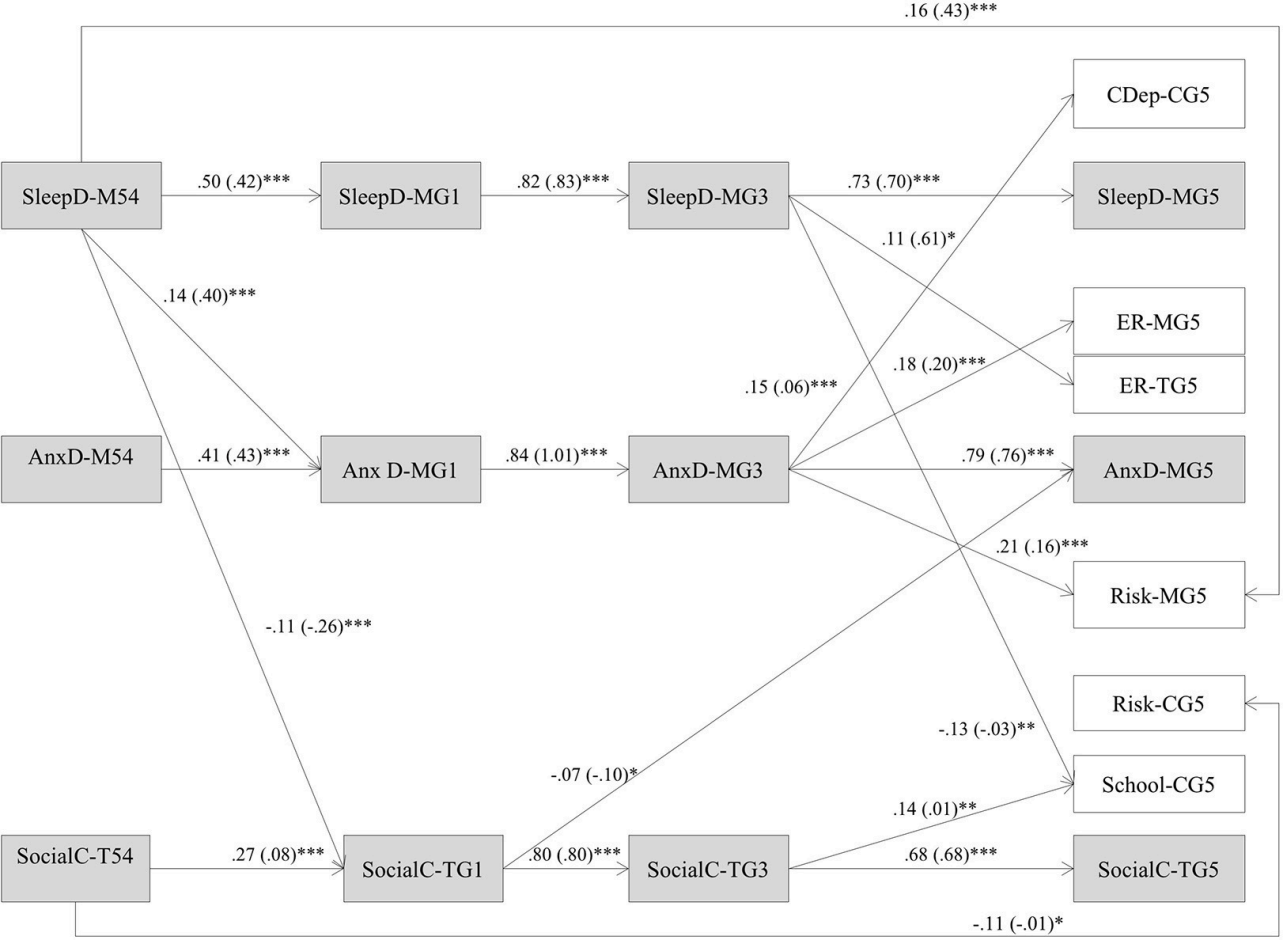
**Girls' final model depicting transactional relations across middle childhood and effects on emotional and social adjustment in preadolescence (***n*** = 529) after controlling for covariates and moderating effects**. Paths for covariance, residual covariance, and covariates, not shown for ease of presentation. Only significant pathways are displayed. Model fit: χ^2^ = 498.163; *df* = 324; *p* = < 0.001; CFI = 0.960; TLI = 0.930; RMSEA [90%] = 0.023 [0.019, 0.026]; Sleep D, sleep problems; AnxD, anxious-depressed symptoms; SocialC, social competence; CDep, child depressive symptoms; School, school competence; ER-M, emotion regulation maternal report; ER-T, emotion regulation teacher report; Risk M, child's risk-taking maternal report; Risk C, child's risk taking child report. ^*^p < 0.05, ^**^p < 0.01, ^***^p < 0.001.

All models provided good overall fit to the data as shown in Table 5. Table 6 presents the direct, overall indirect, and total effects for our final model for the entire sample (Model 5) and for boys and for girls (Model 7) with covariate effects. Because effects extended across four waves of data, we employed the joint significance test (MacKinnon et al., 2002; Haller et al., 2010) to assess the significance of each mediational chain across middle childhood. With this method, a mediational chain is deemed significant if each path in the chain is statistically significant. Our final model reflected only significant structural pathways thus suggesting significance for our overall indirect effects. Figures 2, 3 depict the significant pathways for relations among domains for boys and for girls, respectively, after controlling for covariates and moderating effects. Standardized regression coefficients are shown for each significant pathway with unstandardized regression coefficients shown in parentheses. According to Cohen (1977), cutoff values for small, medium, and large standardized path coefficients are 0.10, 0.30, and 0.50, respectively. When path coefficients were significant both for boys and for girls, tests of critical ratios revealed no significant differences in estimates by gender.

**Table 5:** **Analytic strategy: goodness of fit statistics for each model**.

**Model**	**χ^2^**	**df**	***p***	**CFI**	**TLI**	**RMSEA [90%]**
Model 1: Stability	90.220	36	<0.001	0.982	0.961	0.038 [0.028, 0.048]
Model 2: Bidirectional	47.238	35	0.081	0.996	0.991	0.018 [0.000, 0.030]
Model 3: Add early effects	35.932	33	0.333	0.999	0.998	0.009 [0.000, 0.025]
Model 4: Add adjustment	200.383	83	<0.001	0.969	0.937	0.037 [0.030, 0.043]
Model 5: Add covariates	270.749	145	<0.001	0.972	0.946	0.029 [0.023, 0.034]
Model 6: Gender differences	337.653	182	<0.001	0.958	0.921	0.028 [0.024, 0.033]
Model 7: Add covariates	498.163	324	<0.001	0.960	0.930	0.023 [0.019, 0.026]

*Models 1 through 5 are nested within each other and represent the entire sample (n = 1,057). Models 6 and 7 tested a multiple group model for boys (n = 528) and girls (n = 529) using the same components as Models 4 and 5, respectively*.

**Table 6:** **Direct, overall indirect, and total effects of sleep problems, affective functioning, and emotional and social adjustment at 54 months-of-age, grade 1, grade 3, and grade 5**.

**Path**→**Outcome**			**All Participants**			**Boys**			**Girls**		
			**Direct effects**	**Overall indirect effects**	**Total effects**	**Direct effects**	**Overall indirect effects**	**Total effects**	**Direct effects**	**Overall indirect effects**	**Total effects**
SleepD54	→	AnxDG1	0.14	−	0.14	0.12	−	0.12	0.14	−	0.14
	→	AnxDG3	−	0.11	0.11	−	0.09	0.09	−	0.11	0.11
	→	AnxDG5	−	0.10	0.10	−	0.07	0.07	−	0.08	0.08
	→	SocialCG1	−0.07	−	−0.07	−	−	−	−0.11	−	−0.11
	→	SocialCG3	−	−0.06	−0.06	−	−	−	−	−0.09	−0.09
	→	SocialCG5	−	−0.05	−0.05	−	−	−	−	−0.06	−0.06
	→	School G5	−	−0.05	−0.05	−	−	−	−	−0.06	−0.06
	→	ER-M G5	−	0.03	0.03	−	0.02	0.02	−	0.05	0.05
	→	ER-T G5	−	0.04	0.04	−	−	−	−	0.02	0.02
	→	Risk-M G5	0.14	0.02	0.18	0.15	−	0.15	0.16	0.02	0.18
SleepDG1	→	School G5	−	−0.08	−0.08	−	−	−	−	−0.11	−0.11
	→	ERTG5	−	0.07	0.07	−	−	−	−	0.08	0.08
SleepDG3	→	SchoolG5	−0.11	−	−0.11	−	−	−	−0.13	−	−0.13
	→	ER-TG5	0.10	−	0.10	−	−	−	0.10	−	0.10
AnxD54	→	SleepDG5	0.06	−	0.06	0.13	−	0.13	−	−	−
	→	CDepG5	−	−	−	−	−	−	−	0.05	0.05
	→	ER-MG5	−	−	−	−	0.05	0.05	−	0.06	0.06
	→	Risk-MG5	−	0.04	0.04	−	−	−	−	0.07	0.07
AnxDG1	→	CDepG5	−	−	−	−	−	−	−	0.13	0.13
	→	ER-MG5	−	0.13	0.13	−	0.14	0.14	−	0.15	0.15
	→	Risk-MG5	−	0.12	0.12	−	−	−	−	0.17	0.17
AnxDG3	→	CDep G5	−	−	−	−	−	−	0.14	−	0.14
	→	ER-MG5	0.15	−	0.15	0.17	−	0.17	0.18	−	0.18
	→	Risk-MG5	0.14	−	0.14	−	−	−	0.21	−	0.21
SocialC 54	→	AnxDG5	−0.07	−	−0.07	−0.16	−	−0.16	−	0.02	0.02
	→	School G5	0.12	−	0.12	0.19	−	0.19	−	0.10	0.10
	→	ER-TG5	−	−	−	−	−0.06	−0.06	−	−	−
	→	Risk-M G5	0.16	−	0.16	−0.18	−	−0.18	−	−	−
	→	Risk-C G5	−	−	−	−	−0.08	−0.08	−0.11	−	−0.11
SocialC G1	→	AnxDG5	−	−	−	−	−	−	0.07	−	0.07
	→	SchoolG5	0.10	−	0.10	−	−	−	−	0.10	0.10
	→	ER-TG5	−	−0.12	−0.12	−	−0.14	−0.14	−	−	−
	→	Risk-CG5	−0.18	−	−0.18	−0.18	−	−0.18	−	−	−
SocialC G3	→	SchC G5	0.10	−	0.10	−	−	−	0.12	−	0.12
	→	ER-T G5	−0.15	−	−0.15	−0.17	−	−0.17	−	−	−
NegAffTemp54	→	SleepDG1	−	−	−	0.11	−	0.11	−	−	−
	→	SleepDG3	−	−	−	−	0.07	0.07	−	−	−
	→	SleepDG5	−	−	−	−	0.04	0.04	−	−	−
	→	AnxD G1	0.12	−	0.12	0.14	−	0.14	0.15	−	0.15
	→	AnxDG3	−	0.10	0.10	−	0.11	0.11	−	0.12	0.12
	→	AnxDG5	−	0.09	0.09	−	0.09	0.09	−	0.10	0.10
	→	CDepG5	0.10	−	0.10	0.25	0.01	0.26	−	0.02	0.02
	→	SchoolG5	−	−	−	−0.14	−	−0.14	−	−	−
	→	ER-MG5	0.22	−	0.22	−	−	−	0.32	−	0.32
Slp&Temp.54	→	AnxDG1	−	−	−	0.12	−	0.12	−	−	−
	→	AnxDG3	−	−	−	−	0.10	0.10	−	−	−
	→	AnxDG5	−	−	−	−	0.08	0.08	−	−	−
MDep 1-54	→	SleepDG1	0.09	−	0.09	0.09	−	0.09	−	−	−
	→	SleepDG3	−	0.07	0.07	−	0.07	0.07	−	−	−
	→	SleepDG5	−	0.05	0.05	−	0.04	0.04	−	−	*v*
	→	AnxDG1	0.11	−	0.11	0.11	−	0.11	−	−	−
	→	AnxDG3	−	0.09	0.09	−	0.09	0.09	0.08	−	0.08
	→	AnxDG5	−	0.08	0.08	−	0.07	0.07	−	0.06	0.06
	→	SocialCG1	−0.10	−	−0.10	−	−	−	−0.10	−	−0.10
	→	SocialCG3	−	−0.08	−0.08	−0.13	−	−0.13	−	−0.08	−0.08
	→	SocialCG5	−	−0.07	−0.07	−	−0.11	−0.11	−	−0.05	−0.05
	→	CDepG5	−	0.01	0.01	−	0.01	0.01	−	0.01	0.01
	→	SchoolG5	−	−0.03	−0.03	−	−		−	−0.01	−0.01
	→	ER-TG5	−	−	−	−	0.02	0.02	−	−	−
	→	ER-MG5	0.11	−	0.11	0.14	−	0.14	−	−	−
	→	Risk-MG5	−	0.01	0.01	−	−		−	0.02	0.02
SES 54	→	SocialCG3	0.09	−	0.09	−	−	−	0.11	−	0.11
	→	SocialCG5	−	0.08	0.08	0.15	−	0.15	−	0.08	0.08
	→	Risk-MG5	−0.17	−	−0.17	−0.14	−	−0.14	−0.14	−	−0.14
	→	Risk-CG5	−0.17	−	−0.17	−0.10	−	−0.10	−	−	−
MaritalQ 54	→	AnxDG3	−0.06	−	−0.06	−0.09	−	−0.09	−	−	−
	→	AnxDG5	−	−0.05	−0.05	−	−0.07	−0.07	−	−	−
FirmPCntrl 54	→	AnxDG1	−	−	−	−	−	−	−0.08	−	−0.08
	→	AnxDG3	−	−	−	−	−	−	−	−0.07	−0.07
	→	AnxDG5	−	−	−	−	−	−	−	−0.05	−0.05
	→	CDepG5	−	−	−	−	−	−	−	−0.01	−0.01
	→	ER-MG5	0.08	−	0.08	−	−	−	0.09	−	0.09
	→	Risk-MG5	−	−	−	−	−	−	−	−0.01	−0.01

*Results for cross-domain paths only; Standardized beta weights shown; SleepD, Sleep problems; AnxD, Anxious-depressed symptoms; SocialC, Social competence; CDep, Child Depressive Symptoms; School, School competence; ER-T, Emotion regulation-teacher reported; ER-M, Emotion regulation-mother reported; RiskM, Risk-taking mother reported; RiskC, Risk-taking child reported; NegAffTemp, Negative affective temperament; Slp&Temp, Moderation-Sleep and Temperament; SES, Socioeconomic status; MaritalQ, Marital quality; FirmPCntrl, Firm parental control. All standardized beta weights significant at p < 0.05*.

Results demonstrated several consistent findings for both boys and girls. First, all autoregressive effects were significant indicating continuity over time. Second, cross-lagged results revealed a pathway in which more preschool sleep problems predicted higher rates of anxious-depressed symptoms during middle childhood which in turn predicted more maternal-reported preadolescent emotional reactivity. The direct and indirect effects of anxious-depressed symptoms across middle childhood on preadolescent emotional reactivity were stronger than the effects of preschool sleep. Last, more preschool sleep problems directly predicted higher rates of maternal-reported preadolescent risk-taking 6 years later. For control variables, mothers' reported depressive symptoms were associated with anxious-depressed symptoms, social competence, and several adjustment variables for boys and girls.

For boys, higher rates of preschool anxious-depressed symptoms directly predicted more sleep problems 6 years later in preadolescence. Social competence across middle childhood predicted all preadolescent adjustment domains with the exception of maternal-reported emotional reactivity. Lower levels of *teacher-reported* preschool social competence directly predicted more preadolescent maternal-reported anxious-depressed symptoms, lower self-reported school competence, and more maternal-reported risk-taking. Social competence across middle childhood was negatively associated with *teacher-reported* emotional reactivity and *self-reported* risk-taking. The effects of preschool sleep problems on first grade social competence just missed attainment of statistical significance (*p* = 0.051). Boys' negative affective temperament was positively associated with sleep problems and anxious-depressed symptoms. We found evidence for moderating effects between sleep and anxious-depressed symptoms. The strength of the effect of preschool sleep problems on first grade anxious-depressed symptoms increased as boys exhibited increasingly higher levels of negative affective temperament.

For girls, cross-lagged results revealed several pathways involving sleep problems predicting later functioning. First, more preschool sleep problems predicted *lower teacher-reported* first grade social competence which in turn predicted more maternal-reported preadolescent anxious-depressed symptoms. The indirect effect of preschool sleep on preadolescent anxious-depressed symptoms was small in magnitude. Second, results also revealed a pathway in which more preschool sleep problems predicted more *maternal reported* anxious-depressed symptoms across middle childhood which in turn predicted higher rates of *self-reported* depressive symptoms and maternal-reported risk-taking. Direct and indirect effects of anxious-depressed symptoms on depressive symptoms and risk-taking were stronger in magnitude than the indirect effects of preschool sleep. Third, more sleep problems across middle childhood predicted lower self-reported school competence and higher rates of *teacher-reported* emotional reactivity in preadolescence. Last, social competence in middle childhood negatively predicted two *self-reported* preadolescent adjustment domains—school competence and risk-taking.

## Discussion

We tested a developmental model of sleep and self-regulation across middle childhood to understand the series of transactions that occur for sleep problems, anxious-depressed symptoms, and social competence for boys and girls during a period encompassing two important transitions—the transition to school and the transition to adolescence. Further, we examined the direct and overall indirect effects and pathways of influence to preadolescent emotional and social adjustment. Our study used multiple informants and a four-wave longitudinal design spanning 6 years with a focus on how these effects and pathways might differ for boys and girls. With a large community sample and a research design considered somewhat conservative because of techniques to control for autoregressive effects and covariation among domains (Masten and Cicchetti, 2010), we found that for both boys and for girls, preschool sleep problems predicted anxious-depressed symptoms across middle childhood and preadolescent maternal-reported risk-taking. We conclude that sleep problems during the transition to formal schooling and the transition to adolescence predict affective functioning and preadolescent adjustment, especially in girls. We found minimal evidence for reverse associations. For boys, while sleep also predicted affect and adjustment, social competence played an even more central role in adjustment. Even though effect sizes were small, prior research (Gregory and O'Connor, 2002; Gregory et al., 2009; El-Sheikh et al., 2013; Kelly and El-Sheikh, 2013) and our findings make it clear that the predictive influence of sleep on affective functioning begins early—before formal schooling—and may have long-lasting effects into preadolescence even after considering the influence of important covariates. From a practical perspective, these findings may inform the timing and targeting of intervention because they demonstrate the directionality of effects across middle childhood and pathways of influence to preadolescent adjustment. Our results support Dahl's (1996) and Walker's (2009) theories of sleep as a precipitating factor in affective functioning and Dahl and Conway's (2009) model for the complex overlap in developing regulatory processes involving sleep, affect, and self-regulation.

The developmental course of sleep problems found in this study parallel those of Gregory and O'Connor (2002) and Wang et al. (2016) as evidenced by a gradual decline in sleep problems across middle childhood. Wang et al. in a separate analysis, also identified two subgroups of sleepers—normal sleepers and troubled sleepers—and found a gradual increase in sleep problems in the troubled sleepers' group that commenced around age 10. Even though the authors found no gender differences in the troubled sleepers group, they reported that early emotional difficulties predicted girls' membership in this group but not boys' membership. Our findings add to those of Wang et al. (2016) by demonstrating the presence of gender differences in relations between emotional difficulties and sleep problems in middle childhood but these two studies differ in the direction of effects. In Wang et al. emotional difficulties forecasted sleep problems. In this study, sleep problems predicted emotional difficulties, and sleep problems also predicted preadolescent emotional and social adjustment more so in girls than in boys. The difference in study goals and methodologies may have contributed to alternative findings regarding the direction of effects. Wang et al. used latent trajectory group modeling to uncover discrete subgroups of sleepers over time. In this study, we were interested in the pathways of risk to preadolescent adjustment when sleep problems, emotional difficulties, and social competence were evaluated together and repeatedly across multiple time points using cross-lagged panel analyses. We also studied boys and girls in separate analyses to uncover possible gender differences.

Few studies have examined separate gender models in researching developmental cascades during middle childhood—a period during which pubertal development may begin earlier in girls compared to boys. Gender differences in the effects of sleep on preadolescent adjustment may stem from differences in the timing of pubertal development that alters sleep-wake organization, changes in phase preferences, and increased daytime sleepiness (Carskadon et al., 1980; Laberge et al., 2001). These changes to sleep, combined with the developmental and overlapping changes to self-regulatory processes associated with affect and cognitive control as described by Dahl and Conway's (2009) model, have been linked to risks for compromised psychosocial functioning (Herman-Giddens et al., 1997; Dorn et al., 2006). Several studies have uncovered links between changes to sleep-wake organization and pubertal development between third and fifth grade, particularly in girls (Carskadon et al., 1993; Laberge et al., 2001; Sadeh et al., 2009). Even though this study did not assess pubertal maturation, Susman et al. (2010), who also studied these participants from the NICHD-SECCYD, reported on the timing of pubertal development of secondary sex characteristics in boys and girls during a partly similar timeframe. The authors evaluated Tanner stages by age in boys and girls from 9 ½ to 15 ½ years-of-age. Attainment of Tanner stage 2 is associated with pubertal onset and Tanner stage 5 is associated with the completion of pubertal maturation. Findings indicated that girls, on average, completed Tanner stage 2 between 9.8 and 10.2 years-of-age for all secondary sex characteristics. Boys completed Tanner stage 2 later than girls, on average between 10.3 and 11.3 years-of-age. To revisit the findings of Sadeh et al. (2009) regarding pubertal changes to sleep and physical characteristics, changes to sleep occurred prior to the physical changes associated with puberty. Therefore, our results for girls seem to support a possible link for interactions around the time of pubertal onset (from third to fifth grade) between sleep and anxiety and depressive symptoms to predict preadolescent psychosocial development 2 years later. Because boys reached Tanner stage 2 about 1 year later than girls, it is possible that similar effects between sleep and emotional functioning on preadolescent adjustment may have occurred for boys but took place beyond the timeframe covered by our study.

There were several noteworthy findings related to girls' sleep problems that surfaced when mothers, teachers, and girls reported on affect and adjustment. First, with the exception of girls' self-reported risk-taking behavior, sleep problems at 4 ½ years directly or indirectly predicted emotional and social functioning at every assessment. Second, when mothers of third grade girls reported higher rates of sleep problems, girls reported feeling less competent in school. Our results parallel those of Lemola et al. (2011) who found relations between sleep, optimism, and self-esteem. Third, the only predictor of teachers' report of girls' emotion regulation was mother's reports of sleep problems across the period. Our results align with previous literature that found sleep effects on emotion processing (e.g., Hu et al., 2006; Payne et al., 2008) and support and extend results of Soffer-Dudek et al. (2011) by finding sex differences before adolescence and demonstrating the predictive influence of poor sleep on compromised emotional processing.

None of our covariates were associated with girls' sleep problems at any time across middle childhood. In particular, sleep problems did not appear to be affected by girls' negative affective temperament and mothers' depressive symptoms—two common covariates associated with sleep when using maternal report. We conclude that the persistent influence of girls' sleep problems on affective functioning and adjustment, as reported by multiple informants and controlling for relevant covariates, may signal the beginnings of the interplay between multiple biopsychosocial factors and circadian changes to sleep that are posited to begin in middle childhood, especially for girls (ages 9–10; Sadeh et al., 2009).

In contrast to girls, boys showed little evidence of transactional relations between sleep and affect *across* middle childhood. Instead, three noteworthy patterns surfaced. First, boys' *early* sleep and behavior appeared to play a *direct* role in predicting functioning 6 years later without mediation across middle childhood. When mothers reported higher levels of anxious-depressed symptoms at 4 ½ years in boys, they also reported more sleep problems 6 years later. When teachers reported less competent social skills in 4 ½ year-old boys, mothers reported higher levels of anxious-depressed symptoms 6 years later. These results may suggest that early difficulties with behavioral and emotional self-regulation negatively affect social competence and that poor social competence may interfere with boys' preadolescent difficulties in regulating sleep (e.g., longer sleep onset latency, bedtime ruminations, nightmares) and internalizing problems. Also, it is possible that direct relations between early behaviors and later functioning may have been mediated by unmeasured variables related to sleep such as externalizing behaviors—behaviors that are more prevalent in boys than in girls in this age group (Chen, 2010).

Second, boys' social competence across middle childhood appeared to play a more central role in directly and indirectly predicting four out of six emotional and social adjustment domains in preadolescence compared to girls. Research has shown that girls exhibit better social competence, fewer externalizing behaviors, and more advanced language development during this period. The direct and indirect effects of teacher-reported lower social competence in boys predicted higher rates of teacher-reported emotional reactivity, higher levels of mother-reported and self-reported risk-taking behaviors, and more negative self-reported feelings about their school competence 6 years later. These findings appear to support the competency-based model of development and the findings of Bosquet and Egeland (2006). Failure to achieve competence in one more developmental regulatory processes may lead to internalizing problems and internalizing problems may interfere with attainment of competency.

Third, the influence of covariates on sleep problems was somewhat different for boys than for girls. For boys, but not for girls, we found evidence for the often reported associations for negative affective temperament and maternal depressive symptoms when mothers report boys' sleep problems (Jimmerson, 1991; Minde et al., 1993; Touchette et al., 2007; Weinraub et al., 2012). In fact, boys' negative affective temperament moderated effects between preschool sleep problems and first grade anxiety and depressive symptoms. Specifically, the strength of the effect of preschool sleep problems on first grade anxious-depressed symptoms increased as boys exhibited increasingly higher levels of negative affective temperament. Our results demonstrate the potentiating effects of boys' anxious-depressed symptoms when mothers also reported more sleep disruptions and more fearful, angry, frustrated, and sad behaviors. This early interaction between difficult temperament and sleep problems continued to exert indirect effects on boys' anxious and depressed behaviors throughout middle childhood and preadolescence.

Few studies consider separate gender models in researching developmental cascades during middle childhood—a period during which pubertal development may begin earlier in girls compared to boys. Finding different models predicting preadolescent adjustment in boys and girls may stem from several factors. First, early phases of pubertal development may alter sleep-wake organization leading to changes in phase preferences and increased daytime sleepiness (Carskadon et al., 1980; Laberge et al., 2001). These changes have been linked to risks for compromised psychosocial functioning (Herman-Giddens et al., 1997; Dorn et al., 2006). Several studies have uncovered links between changes to sleep-wake organization and pubertal development between third and fifth grade, particularly in girls (Carskadon et al., 1993; Laberge et al., 2001; Sadeh et al., 2009).

We revisit Carskadon's (2011) metaphor of “the perfect storm” and Dahl and Conway's (2009) model of self-regulation to explicate our findings about the cumulative effects of relations found across middle childhood involving sleep, affect, and social competence on girls' emotional and social adjustment during the transition to adolescence. The “storm” represents the convergence of changes to multiple biopsychosocial regulatory systems that conspire to reduce sleep with risk for dysregulation of physical, emotional, and social functioning. This study found that the cumulative effects of mothers' reported sleep problems predicted almost all emotional and social adjustment domains (using multiple informants) in preadolescent girls. We found that both constitutional and contextual covariates were unassociated with girls' sleep problems. Perhaps our findings herald “a rolling thunder” in middle childhood in girls that precedes the “perfect storm” that comes later with adolescence.

We highlight two additional findings from this study. For both boys and girls, preschool sleep problems *directly* predicted maternal-reported preadolescent risk-taking. Our findings support those of O'Brien and Mindell (2005) and Thomas et al. (2015) and add to the literature by uncovering the predictive influence of preschool sleep on risk-taking 6 years later. It is possible that unmeasured variables, such as externalizing behaviors or working memory processes, may have mediated this relation (Boyer, 2006; Chen, 2010; Thomas et al., 2015). Nonetheless, relations between early sleep problems and preadolescent risk-taking were among the strongest found in this study for sleep predicting affect or adjustment. Difficulties regulating sleep early in middle childhood may be linked to a lack of self-control that, by preadolescence, manifests itself in making poor choices and an inability to inhibit responses associated with risky behavior.

To our knowledge, this study is among the first (see also Vaughn et al., 2015) to establish longitudinal relations between sleep problems and social functioning in middle childhood using both mother and teacher reports. This finding suggests that sleep difficulties during critical periods of brain development may interfere with the development of neural mechanisms that govern affective regulation (Marks et al., 1995; Dahl, 1996; Dahl and Conway, 2009; Ringli and Huber, 2011; Kurth et al., 2012). Social competence relies on the simultaneous and highly complex cognitive and emotional processing that is most sensitive to sleep loss. At the same time, social *development* depends, in part, on experiential interactions with peers who possess strong social skills (Hay et al., 2004; Wentzel, 2009). Our findings for persistent *indirect* effects of preschool sleep problems on social competence across middle childhood may be explained by early social deficiencies that continue to restrict relations with more socially proficient peers that, in turn, may compromise children's adjustment.

### Limitations

Reliance on maternal report rather than more objectives measures of polysomnography or actigraphy represented a considerable limitation in this study and introduced the potential for shared reporter variance to influence some of our findings; however, our analyses controlled for mothers' report of their own depressive symptoms and children's temperament, two factors found to predict children's sleep problems and anxious-depressed symptoms. The use of child and teacher reports as outcome measures and teachers' reports for measures of social competence also help extend these findings beyond the limits of maternal report (Podsakoff et al., 2003). Nevertheless, our findings using maternal reports are similar to those of Kelly and El-Sheikh (2013) using more objective measures of sleep. Thus, it may be reasonable to consider the importance of these findings despite the potential for shared rater bias. Our large sample (*n* = 1,057) enabled us to conduct complex and rigorous analyses using four waves of data with a developmental cascade model, but cost and time factors limited the collection of more objective assessments of sleep with this large sample (Becker et al., 2015).

### Practical implications

These findings suggest that elevated levels of sleep problems and deficits in affective functioning as children begin formal schooling may have implications for children's school success and optimal development. For girls, early and middle childhood sleep problems influenced adjustment at the end of middle childhood, more so than boys, suggesting there may be developmental differences in relations between sleep and pubertal development. Thus, parents and clinicians may want to pay closer attention to girls' sleep in relation to emotional behavior and social adjustment throughout middle childhood. For boys, because mothers reported more sleep problems in boys with higher levels of negative affectivity and also reported more depressive symptoms in themselves, researchers may want to examine these interrelations more closely for families with boys.

In their review of the efficacy of behavioral treatments for bedtime problems and night wakings in early childhood, Mindell et al. (2006) found that over 90% of behavioral interventions produced reliable and durable improvements in children's sleep problems. These findings are encouraging because they suggest that behavioral treatments, including preventive parent education, may alter the course of early maladaptive patterns that may lead to future psychosocial disorders in typically developing children.

## Ethics statement

This study was carried out in accordance with the recommendations of the Institutional Review Board (IRB) of Temple University with written informed consent from all subjects. The protocol was approved by the IRB of Temple University.

## Author contributions

Conception or design of work: JF and MW. Data collection: MW. Data analysis: JF. Data interpretation: JF and MW. Drafting the article: JF. Critical revision of the article: JF and MW. Final approval of the version to be published: JF and MW.

## Funding

This project was partially funded by NICHD through a cooperative agreement (5 U10 HD027040) to MW, Temple University.

### Conflict of interest statement

The authors declare that the research was conducted in the absence of any commercial or financial relationships that could be construed as a potential conflict of interest.
